# Marked improvement in autoimmune pulmonary alveolar proteinosis with severe hypoxemia in a patient treated with ambroxol: a case report

**DOI:** 10.1186/s13256-015-0588-6

**Published:** 2015-05-06

**Authors:** Nao Oda, Koji Tamai, Yujiro Suzuki, Harukazu Yoshimatsu, Hirofumi Matsuoka, Yusuke Matsumoto, Nobuhiko Okada

**Affiliations:** Department of Respiratory Medicine, Shinko Hospital, 1-4-47, Wakihama-cho, Chuo-ku Kobe, 651-0072 Japan

**Keywords:** Autoimmune pulmonary alveolar proteinosis, Ambroxol, Severe hypoxemia

## Abstract

**Introduction:**

Pulmonary alveolar proteinosis is characterized by accumulation of surfactant and phospholipids in the pulmonary alveoli. Whole lung lavage is considered the first-line therapy, which requires special techniques. To the best of our knowledge, there have only been limited reports that have demonstrated the effectiveness of ambroxol on a mild case of pulmonary alveolar proteinosis.

**Case presentation:**

A 72-year-old Japanese woman presented to our hospital with a one-year history of productive cough and progressive dyspnea. Her chest computed tomography scan showed a bilateral crazy-paving pattern in both of her lungs. She was diagnosed with autoimmune pulmonary alveolar proteinosis based on bronchoalveolar lavage findings and the presence of serum anti-granulocyte macrophage colony-stimulating factor antibodies. She was severely hypoxemic, so we recommended whole lung lavage or inhaled granulocyte macrophage colony-stimulating factor treatment, which she refused. We initiated treatment with ambroxol and her symptoms markedly improved.

**Conclusions:**

Although whole lung lavage is the first-line therapy for pulmonary alveolar proteinosis, oral ambroxol could be an alternative treatment option, even in patients with severe respiratory compromise.

## Introduction

Pulmonary alveolar proteinosis (PAP) is a rare interstitial disease caused by accumulation of surfactant and phospholipids in the pulmonary alveoli, resulting in dyspnea, cough, fatigue, and a crazy-paving pattern on chest computed tomography (CT) [1]. PAP is classified as autoimmune or non-autoimmune, including secondary and congenital PAP; more than 90% of cases are diagnosed as autoimmune PAP [2]. The pathogenesis of autoimmune PAP involves a neutralizing antibody that directly binds to granulocyte-macrophage colony-stimulating factor (GM-CSF) and blocks its binding to GM-CSF receptors on cells, which leads to inhibition of signaling, thereby inhibiting surfactant maturation [3]. Although whole lung lavage (WLL) remains the current standard of care for PAP, other therapies, including inhaled GM-CSF, rituximab, and plasmapheresis [4,5] have been proposed. A few studies have reported that ambroxol, a commonly used expectorant, can relieve PAP [6,7]. Here, we report a case of autoimmune PAP with chronic severe hypoxemia, which responded to oral administration of ambroxol.

## Case presentation

**Figure 1:**
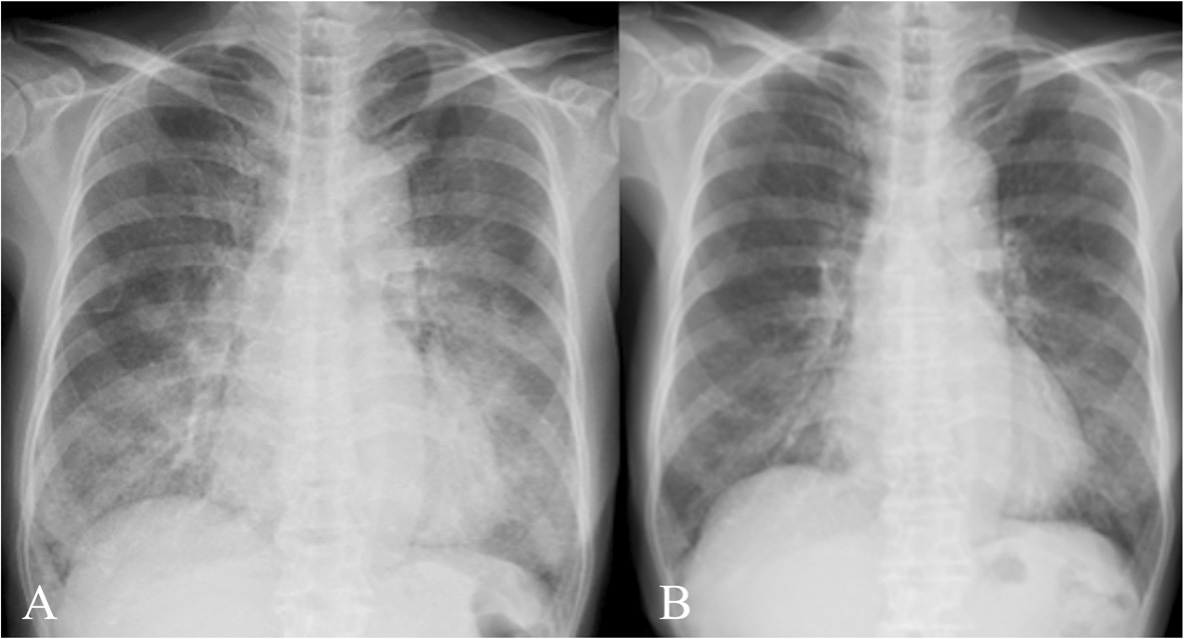
Chest radiographs before and after the treatment. **(A)** Chest radiographs (CXR) on admission. **(B)** CXR findings improved after one month of treatment with ambroxol.

**Figure 2:**
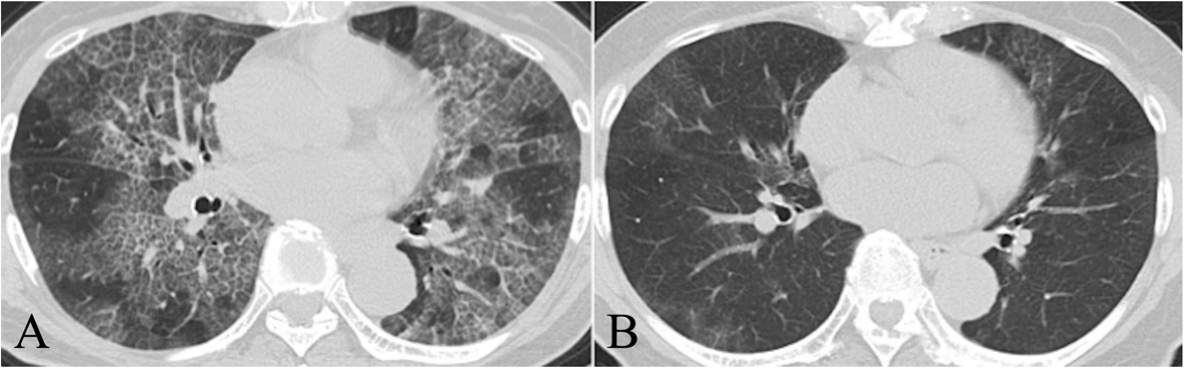
Chest computed tomography (CT) scan before and after the treatment. **(A)** Chest CT scan on admission. **(B)** Chest CT scan after five months of treatment with ambroxol showed almost no abnormal shadows.

A 72-year-old Japanese woman presented to our hospital with a one-year history of productive cough and progressive dyspnea on exertion. Her past medical history included only osteomyelitis at 35-years-old and she was not taking any medications. She had never smoked or inhaled dust as an occupational hazard. Her vital signs were as follows: body temperature of 36.6°C, blood pressure of 130/80mmHg, heart rate of 67 beats/min, and SpO_2_ level of 80% (room air). Her arterial blood gas analysis revealed a pH of 7.44, PaO_2_ level of 43.2mmHg, PaCO_2_ level of 39.2mmHg, and HCO_3_^−^ level of 26.4mEq/L on room air. Her chest radiographs (CXR) showed bilateral infiltrates in her mid and lower lung zones (Figure 1A), and her chest CT scan showed bilateral ground glass opacities with thickened interlobular septa, an appearance known as the ‘crazy-paving’ pattern (Figure 2A).

Her laboratory tests revealed a normal complete blood count, high lactate dehydrogenase (543/mL), and high Krebs von den Lungen-6 level (16,189U/mL). Her pulmonary function tests taken on admission revealed a vital capacity (VC) of 1.49L, %VC of 68.6%, forced expiratory volume in one second (FEV_1_) of 1.17L, FEV_1_/forced vital capacity (FVC) predicted ratio of 76.0%, and FEV_1_/FVC actual ratio of 84.2%. We retrieved 26/100mL of bronchoalveolar lavage fluid (BALF), which was milky in appearance. Her BALF total cell count was 6.0×10^4^/mL; cell differentiation revealed 23% neutrophils, 1% eosinophils, 17% lymphocytes, and 59% macrophages. Her BALF contained copious eosinophilic, periodic acid Schiff-positive granular material, a finding that supported a diagnosis of PAP. In addition, a high level of anti GM-CSF antibody was detected in her serum (57.5μg/mL), leading to a diagnosis of autoimmune PAP. Her disease severity score (DSS) was classified as DSS 5, based on severe hypoxemia (PaO_2_ level of 43.2mmHg).

Considering her severe DSS, we recommended treatment with WLL under general anesthesia or inhaled GM-CSF therapy. However, she refused both treatments because of concern about complications and medical costs; she also refused hospitalization. Therefore, we started domiciliary oxygen therapy and an alternative treatment of 45mg/day of ambroxol (TEIJIN PHARMA, Tokyo, Japan). She was discharged, with frequent scheduled checkup examinations at our outpatient clinic. A few days after starting ambroxol, she showed loosened phlegm and reduced cough, with progressively improving dyspnea. Her oxygen saturation level in room air rose to 95%, and her CXR results showed improvement after one month (Figure 1B). Her chest CT scan after five months of treatment showed almost no abnormal shadows (Figure 2B), and her pulmonary function was improved (VC of 1.88L and %VC of 90.4%). She has continued ambroxol treatment for 17 months without disease progression.

## Discussion

PAP is a rare lung disease characterized by the intra-alveolar accumulation of surfactant lipids and proteins, which impairs gas exchange and results in respiratory failure [3]. The majority of PAP is autoimmune PAP. Dranoff *et al*. found that an absence of local GM-CSF-dependent activation of macrophage in the lungs is involved in surfactant clearance [8]. Autoimmune PAP is now considered to occur via a neutralizing antibody, which inhibits GM-CSF binding to receptors [3]. This leads to a defect of alveolar macrophages and abnormal surfactant metabolism, which results in intra-alveolar accumulation of surfactant. WLL, the standard therapy for PAP, has beneficial effects in approximately two thirds of patients, however, it requires special techniques and carries the risk of complications, including hypoxemia, hydropneumothorax, acute respiratory distress syndrome, and post-procedure infections [1]. Inhaled GM-CSF has also been reported as effective therapy; Tazawa *et al*. conducted a phase II trial of GM-CSF therapy for PAP [9]. Of 35 patients completing GM-CSF therapy, 24 improved, resulting in an overall response rate of 62% (24 out of 39; intension-to-treat analysis). The safety and efficacy of rituximab and plasmapheresis in PAP patients is now being studied [1]. Our patient was a candidate for WLL and inhaled GM-CSF treatment because she had severe hypoxemia and was classified as DSS 5 [1]. However, she refused our recommendations because she feared complications and medical costs, so we chose conservative treatment with ambroxol.

The potential effects of ambroxol for PAP can be evaluated based only on limited case reports, because controlled trials have not been conducted. Diaz *et al*. first reported the use of ambroxol in a PAP patient in 1984, with inhalation of 30mg of ambroxol every six hours, leading to resolution of symptoms and improvement in CXR findings and pulmonary function tests [7]. However, details about the patient’s characteristics were not described. Only three additional articles about the efficacy of ambroxol for PAP are available in the PubMed database in English. Hashizume described improvement in a patient with mild PAP (PaO_2_ level of 61.3mmHg versus 43.2mmHg in our patient) treated with 45mg/day of ambroxol [6]. Mahut *et al*. described a pediatric patient who experienced a 10-year remission from PAP with ambroxol treatment, but the report concluded that the remission might have been spontaneous [10]. Garcia *et al*. reported the unsuccessful treatment of two PAP patients with oral ambroxol [11]. It remains unclear which patients will benefit from ambroxol and how ambroxol affects the pathophysiology of PAP. Ambroxol may act on type II pneumocytes, which leads to chemicophysical and functional changes in alveolar macrophages, probably through an improvement in surfactant secretion and uptake system [12]. We hypothesize that this may improve PAP.

Our patient was successfully treated with oral ambroxol, despite the severity of her disease. Though WLL or inhaled GM-CSF therapy remains the first-line therapy for PAP, oral ambroxol could be an alternative treatment option for patients with PAP who have severe respiratory compromise, especially if WLL and inhaled GM-CSF therapy is not feasible, as it is a simple treatment and with few side effects.

Spontaneous resolution occurs in an estimated 7.9% of PAP patients [13]. However, spontaneous resolution in patients with a PaO_2_ level of less than 60mmHg is considered very rare [6]. Our patient had a PaO_2_ level of 43.2mmHg and her symptoms had been worsening for more than a year leading to a diagnosis at the most severe stage of the disease. Furthermore, her symptoms immediately improved after initiating ambroxol. Hence, we reasonably conclude that ambroxol, not spontaneous resolution, contributed to the relief of her PAP.

## Conclusions

Ambroxol can improve PAP, even in patients with severe respiratory compromise. Although WLL is the first-line therapy for PAP, oral administration of ambroxol should also be considered.

## Consent

Written informed consent was obtained from the patient for publication of this case report and accompanying images. A copy of the written consent is available for review by the Editor-in-Chief of this journal.

## Abbreviations

BALF: Bronchoalveolar lavage fluid
CT: Computed tomography
CXR: Chest radiographs
DSS: Disease severity score
FEV_1_: Forced expiratory volume in one second
GM-CSF: Granulocyte-macrophage colony-stimulating factor
PAP: Pulmonary alveolar proteinosis
VC: Vital capacity
WLL: Whole lung lavage
